# Le léiomyosarcome de la vessie: à propos de 3 cas

**DOI:** 10.11604/pamj.2018.30.19.10160

**Published:** 2018-05-09

**Authors:** Kadouri Youssef, Ousmane Nago Dembele, Ziani Idriss, Hachem Elsayegh, Ali Iken, Lounis Benslimane, Yassine Nouini

**Affiliations:** 1 Service d’Urologie A, CHU IBN Sina, Rabat, Maroc

**Keywords:** Vessie, tumeur rare, léiomyosarcome, Bladder, rare tumor, leiomyosarcoma

## Abstract

Le léiomyosarcome de la vessie est une tumeur rare hautement maligne. Il survient aussi bien chez l'enfant que chez l'adulte avec une incidence maximale au-delà de 60 ans. Il semble atteindre préférentiellement le sexe masculin avec un sexe ratio 3/1. Sa présentation clinique est non spécifique et dominée par l'hématurie. La résection endoscopique de la vessie avec un examen anathomopathologique permet de poser le diagnostic. Le traitement du léiomyosarcome de la vessie reste sujet à de nombreuses controverses du fait de la rareté des cas rapportés dans la littérature. Cependant le traitement de choix semble être une cystectomie totale précédée d'une chimiothérapie néo-adjuvante lorsque l'état général du patient le permet. Nous rapportons une série de 3 cas de léiomyosarcome vésical qui ont été traités tous par une cystectomie totale avec des suites opératoires simple.

## Abstract (en)

Bladder leiomyosarcoma is a rare highly malignant tumor. It can occur in children and adults with a maximum incidence in patients over 60 years of age. It mainly affects men, with a sex ratio of 3/1. Its clinical features are non-specific and dominated by the haematuria. Endoscopic resection of the bladder associated with anatomo-pathological examination allow the diagnosis. Its treatment remains controversial due to the rarity of cases reported in the literature. Neoadjuvant chemotherapy followed by radical cystectomy is the treatment of choice, if the patient's clinical condition permit. We report 3 cases of bladder leiomyosarcoma treated by radical cystectomy and with uneventful postoperative course.

## Introduction

Le léiomyosarcome est une tumeur mésenchymateuse maligne avec différenciation musculaire lisse. Sa localisation vésicale est rare, estimée entre 0,38 et 0,64% de toutes les tumeurs vésicale [1-3]. Il peut toucher aussi bien l'enfant que l'adulte avec une incidence maximale au-delà de 60 ans. Il semble atteindre préférentiellement le sexe masculin avec un sexe ratio à 3/1 [1, 4]. Le but de notre travail est d'étudier les particularités épidémiologiques, cliniques, radiologiques, thérapeutiques et évolutives de ce type de tumeur.

## Méthodes

Nous rapportons une série de trois cas de léiomyosarcome vésical, colligés au service d'urologie A du CHU Ibn Sina entre 2007 et 2015. Les données recueillies pour la réalisation de ce travail proviennent des dossiers des patients dans les archives du service et des comptes rendus opératoires.

## Résultats

**Figure 1:**
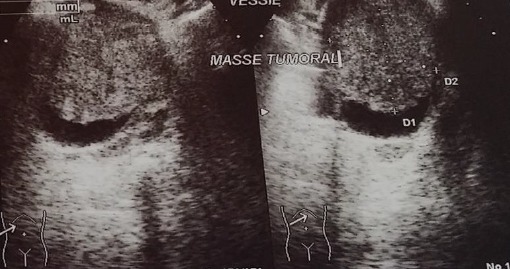
Echographie de la patiente N°3: objectivant le processus intra-vésical

**Figure 2:**
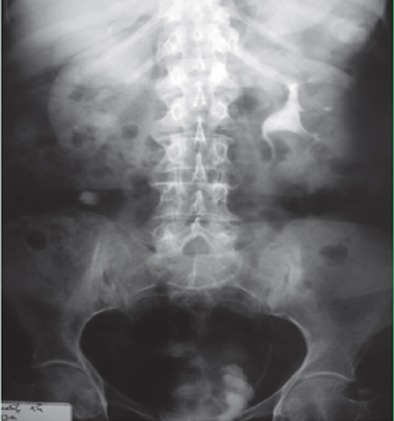
UIV tardif de 1h de la patiente N°2: image lacunaire amputant la corne vésicale droite et le dôme vésical avec urétéro-hydronéphrose gauche

**Figure 3:**
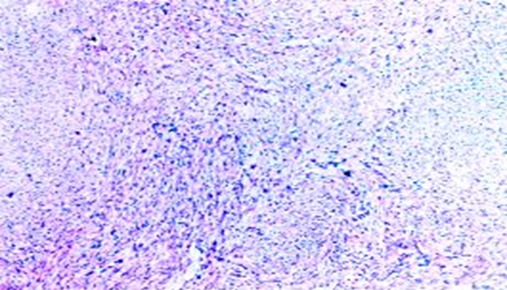
Prolifération à forte densité cellulaire, faite de cellules fusiformes qui forment des faisceaux perpendiculaires (HES X 40)

Les caractéristiques des patientes ont été détaillées dans le Tableau 1. Il s'agit de trois femmes, l'âge moyen était de 65 ans avec des extrêmes allant de 50 ans à 80 ans. Le maitre symptôme était une hématurie totale caillotante, associé dans deux cas à un syndrome irritatif du bas appareil urinaire (une pollakiurie et des brûlures mictionnelles) et une AEG avec anorexie et amaigrissement non chiffré dans un seul cas. L'examen physique était normal chez deux patientes tandis que chez une 3eme a objectivé un plancher vésical et une paroi vaginale antérieure infiltrées avec une masse pelvienne palpable. Sur le plan paraclinique, deux patientes ont été examinés par une échographie réno-vésicale, et une patiente par une urographie intraveineuse. L'échographie avait permis d'explorer la morphologie vésicale et de suspecter le diagnostic d'une tumeur de vessie en objectivant une masse solide, échogène, discrètement hétérogène au niveau du trigone chez la patiente N 1 et un processus de la paroi antérieure de la vessie de 58*36 mm chez la patiente N 3 (Figure 1), tandis que l'UIV avait montré une image lacunaire amputant la corne vésicale droite et le dôme vésical avec urétéro-hydronéphrose gauche chez la patiente N 2 (Figure 2). La résection trans-urétrale de la vessie (RTUV), sur des urines stériles, était complète dans deux cas et incomplète dans un seul cas. L'examen anatomopathologique des copeaux de résection avec l'étude immuno-histochimique ont permis de confirmer le diagnostic. Le scanner thoraco-abdomino-pelvien dans le cadre du bilan d'extension a été réalisé chez les 3 patientes et il n'a pas objectivé de localisation secondaire. Les patientes ont subi donc une pelvectomie antérieure avec curage ganglionnaire bilatérale. L'examen anatomopathologique des pièces opératoires confirmait le diagnostic de léiomyosarcome vésical (Figure 3). Les suites opératoires ont été simples.

**Tableau 1:** Récapitulatif des cas cliniques de notre série

	Patiente N°1 Madame H.H	Patiente N°2 Madame K.I	Patiente N°3 Madame B.H
**Age**	50 ans	80 ans	65 ans
**Circonstances de découvertes**	Hématurie totale caillotante + sd irritatif du bas appareil urinaire	Hématurie totale caillotante + sd irritatif du bas appareil urinaire	Hématurie totale caillotante
**Antécédents**	Pas de notion de tabagisme Pas ATCD médico-chirurgicaux	Cholécystectomie à l’âge de 59 Taille vésicale pour lithiase de vessie à l’âge de 69 Pas de tabagisme	Appendicectomie à l’âge de 49 Pas de notion de tabagisme
**Examen clinique**	Normal	Plancher vésical et paroi vaginale antérieure infiltrés Masse dure au niveau pelvien et de la FID	Normal
**Examens paracliniques**	Echographie réno-vésicale:masse solide, échogène, discrètement hétérogène au niveau du trigone sans retentissement sur le haut appareil urinaire Bilan biologique normal	UIV: image lacunaire amputant la corne vésicale droite et le dôme vésical avec urétéro-hydronéphrose gauche Bilan biologique: anémie à 6,7 avec insuffisance rénale	Echographie réno-vésicale:unprocessus de la paroi antérieurede la vessie de 58*36 mm Bilan biologique normal
**RTUV + Anapath**	Complete et profonde Anapath: léiomyosarcome vésical	1^ère^: complète et profonde Anapath: pseudo sarcome vésical 2^ème^: léiomyosarcome vésical	Incomplète et profonde Anapath: léiomyosarcome dehaut grade infiltrant et ulcérantla muqueuse
**Bilan d’extension**	TDM TAP: pas de localisation secondaire	TDM TAP: processustissulaire vésical infiltrant la paroi abdominale antérieure sans localisation secondaire	TDM TAP: pas de localisationsecondaire
**Traitement**	Pelvectomie antérieure	Pelvectomie antérieure	Pelvectomie antérieure
**Examen anatomopathologique de la pièce opératoire**	léiomyosarcome de haut grade infiltrant la paroi vésicale sans atteinte du tissu péri-vésical avec marge négative, absence de métastases ganglionnaires	léiomyosarcome vésical de grade 1 de la FNCLCC, les recoupes urétérales, l’utérus, les ovaires et collerette vaginale sont indemnes, absence de métastases ganglionnaires	léiomyosarcome de haut grade infiltrant la paroi vésicale sans atteinte du tissu péri-vésical avec marge négative, absence de métastases ganglionnaires
**Suites**	Simple	Simple	Simple

## Discussion

Le léiomyosarcome est une tumeur ubiquitaire des tissus mous qui peut toucher tout le tractus uro-génital, sa localisation au niveau de la vessie est extrêmement rare, son incidence est estimée à moins de 1% de toutes les tumeurs de vessie. Le mécanisme de genèse est inconnu, toutefois plusieurs facteurs pathogéniques ont été rapportés dans la littérature comme la transformation maligne d'un léiomyome en léiomyosarcome [5], l'irritation chronique de la vessie, les radiations [6], certains carcinogènes chimiques, la mutation du gène du rétinoblastome, des facteurs héréditaires dans 1% des cas (Syndrome de Li-Fraumeni, mutation du gène NF-1 et mutation du gène Rb-1) ainsi qu'une chimiothérapie au long cours par le cyclophosphamide [7], La symptomatologie clinique est dominée par les symptômes urinaires, principalement une hématurie macroscopique souvent massive dans 75% [8] des cas et peut être isolée ou associée à des signes d'irritation vésicale ou à une masse hypogastrique dans les formes avancées comme le cas de notre 2^ème^ patiente. Sur le plan radiologique, le léiomyosarcome vésical ne présente pas d'images caractéristiques. La cystoscopie permet la visualisation de la tumeur et sa biopsie, cette tumeur prend généralement un aspect ovoïde ou polypoïde, encapsulée ou non, se développant volontiers à proximité des méats urétéraux expliquant le retentissement précoce sur le haut appareil [2]. Elle prend naissance au niveau de la musculeuse vésicale pour s'étendre ensuite vers la muqueuse qu'elle refoule avant de l'envahir. Il s'agit d'une tumeur dotée d'un important potentiel d'extension locale qui survient avant les métastases [9]. Le diagnostic positif est posé par l'examen anatomo-pathologique qui montre une prolifération maligne à cellules fusiformes et renseigne sur l'index mitotique ainsi que l'immuno-histochimie qui montre un marquage fortement positif par les anticorps anti actine musculaire lisse. Le traitement du léiomyosarcome de la vessie reste sujet à de nombreuses controverses du fait de la rareté des cas rapportés d'où l'intérêt d'une approche thérapeutique multidisciplinaire dans un centre spécialisé en sarcomes. La chirurgie reste le seul traitement curateur dont l'objectif est le contrôle local maximum avec marges larges (> 2 cm) [10-12] en essayant de préserver la fonction avec un minimum de séquelles. R0: pas de résidus (rechute < 20%); R1: résidus microscopiques (rechute > 50%; R2: résidus macroscopiques (rechute 100%). Certains auteurs [13-15] proposent une cystectomie partielle pour les tumeurs localisées, ne dépassant pas 5 cm, bien que SEEN [16] rapporte 100% de récidive tumorale après ce traitement conservateur. Ainsi, la majorité des auteurs préconisent une cystectomie totale [17]. ALABASTER [18] propose une uréthrectomie systématique du fait de la grande fréquence des localisations uréthrales concomitantes ou secondaires. Si la chirurgie d'emblée ne peut être R0, une chimiothérapie doit être proposée pour tenter une résection carcinologique en cas de réponse et minimiser les séquelles. La chimiothérapie adjudante n'est pas un standard mais peut être discutée en cas de facteurs de mauvais pronostiques tels que la taille et le grade histologique. Concernant la place de la radiothérapie, elle est un standard en adjuvant à la chirurgie si la tumeur est de haut grade (2-3), de taille > 5 cm ou si les marges sont R1-R2 sans possibilité de reprise [19]. L'apport de la radio-chimiothérapie a permis d'améliorer le pronostic du léiomyosarcome vésical, avec un taux de survie sans récidive qui peut attendre 3 à 8 ans selon une série AHLERING [9] rapporté sur 7 cas de léiomyosarcome vésical. Selon une autre étude rétrospective réalisée dans le Centre d'Oncologie MD Anderson sur 36 cas, le taux de survie à 1 an, 3 ans et, 5 ans est respectivement de 88,6% et de 62% [20].

## Conclusion

Le léiomyosarcome de la vessie est une tumeur rare et hautement maligne. Seul l'examen anatomo-pathologique permet de porter le diagnostic positif. Le traitement de choix semble être une cystectomie totale précédée d'une chimiothérapie néo-adjuvante lorsque l'état général du patient le permet.

## Conflits d’intérêts

Les auteurs ne déclarent aucun conflit d'interêt.
